# Impact of diabetes, obesity and hypertension on preterm birth: Population-based study

**DOI:** 10.1371/journal.pone.0228743

**Published:** 2020-03-25

**Authors:** Howard Berger, Nir Melamed, Beth Murray Davis, Haroon Hasan, Karizma Mawjee, Jon Barrett, Sarah D. McDonald, Michael Geary, Joel G. Ray

**Affiliations:** 1 Division of Maternal-Fetal Medicine, Department of Obstetrics and Gynecology, St. Michael’s Hospital, University of Toronto, Toronto, Ontario, Canada; 2 Division of Maternal-Fetal Medicine, Department of Obstetrics and Gynecology, Sunnybrook Health Sciences Centre, University of Toronto, Toronto, Ontario, Canada; 3 Departments of Obstetrics and Gynecology, Midwifery Education Program, McMaster University, Hamilton, Ontario, Canada; 4 Better Outcomes Registry & Network (BORN) Ontario, Children’s Hospital of Eastern Ontario (CHEO), Ottawa, Ontario, Canada; 5 Division of Maternal-Fetal Medicine, Departments of Obstetrics and Gynecology, Radiology and Health Research Methods, Evidence & Impact, McMaster University, Hamilton, Ontario, Canada; 6 Department of Obstetrics & Gynaecology, Rotunda Hospital, Rotunda, Dublin 1, Ireland; 7 Departments of Medicine and Obstetrics and Gynaecology, St. Michael’s Hospital, University of Toronto, Toronto, Ontario, Canada; Universita degli Studi dell'Insubria, ITALY

## Abstract

**Objective:**

To determine the impact of pre-pregnancy diabetes mellitus (D), obesity (O) and chronic hypertension (H) on preterm birth (PTB).

**Methods:**

Retrospective population-based cohort study in Ontario, Canada between 2012–2016. Women who had a singleton livebirth or stillbirth at > 20 weeks gestation were included in the cohort. Exposures of interest were D, O and H, individually, and in various combinations. The primary outcome was PTB at 24^1/7^ to 36^6/7^ weeks. PTB was further analyzed by spontaneous or provider-initiated, early (< 34 weeks) or late (34–37 weeks), and the co-presence of preeclampsia, large for gestational age (LGA), and small for gestational age (SGA). Multivariable Poisson regression models with robust error variance were used to generate relative risks (RR), further adjusted for maternal age and parity (aRR). Population attributable fractions (PAF) were calculated for each of the outcomes by exposure state.

**Results:**

506,483 women were eligible for analysis. 30,139 pregnancies (6.0%) were complicated by PTB < 37 weeks, of which 7375 (24.5%) had D or O or H. Relative to women without D or O or H, the aRR for PTB < 37 weeks was higher for D (3.51; 95% CI 3.26–3.78) and H (3.81; 95% CI 3.55–4.10) than O (1.14; 95% CI 1.10–1.17). The combined state of DH was associated with a significantly higher aRR of PTB < 37 weeks (6.34; 95% CI 5.14–7.80) and < 34 weeks (aRR 10.33, 95% CI 6.96–15.33) than D alone. The risk of provider initiated PTB was generally higher than that for spontaneous PTB. Pre-pregnancy hypertension was associated with the highest risk for PTB with preeclampsia (aRR 45.42, 95% CI 39.69–51.99) and PTB with SGA (aRR 9.78, 95% CI 7.81–12.26) while pre-pregnancy diabetes was associated with increased risk for PTB with LGA (aRR 28.85, 95% CI 24.65–33.76).

**Conclusion:**

Combinations of DOH significantly magnify the risk of PTB, especially provider initiated PTB, and PTB with altered fetal growth or preeclampsia.

## Introduction

The rising global burden of diabetes mellitus (D), obesity (O) and chronic hypertension (H) has a significant impact on maternal-fetal health [1]. It is estimated that one in five pregnancies are affected by one or more of these conditions [2]. Individually, D, O or H are each associated with significant perinatal morbidity and mortality, largely mediated by an increased risk of stillbirth, the hypertensive disorders of pregnancy, abnormal fetal growth and preterm birth (PTB) [3–5].

Spontaneous (sPTB) and provider-initiated (piPTB) PTB are the leading cause of neonatal mortality and morbidity globally[6]. While the risk of PTB associated with D, O or H has been described previously[7–9], there is a paucity of data regarding their combined contribution, including the associated risk of early and late PTB, as well as piPTB and sPTB. A better understanding of the magnitude of the contribution of D, O and H to the different forms of PTB can assist in global efforts to devise prevention and intervention strategies.

The current study was undertaken to identify the risk of PTB in relation to D, O and/or H, as well as different types and degrees of PTB, including that with abnormal fetal growth and maternal preeclampsia. This study generated absolute and relative risks (RR), as well as population attributable fractions (PAF) for PTB that could potentially guide preventive efforts aimed at reducing D, O and H in women of reproductive age, as well as the burden of PTB once pregnant.

## Materials and methods

### Study design and population

This retrospective population-based cohort study included all women who had a singleton hospital birth at > 20 weeks’ gestation in Ontario, Canada, April 1, 2012 and March 31, 2016.

### Setting

Ontario is the most populous province in Canada, comprising roughly 40% of the entire Canadian population[10]. All permanent residents of Ontario receive universal health coverage under the government-funded provincial health insurance plan (OHIP).

### Exposure

The exposures of interest were the exclusive D, O and H states individually, and in all exclusive combinations(DO, DH, OH and DOH).

### Data sources

Women were identified using the Better Outcomes Registry & Network (BORN) information system (BIS) (https://www.bornontario.ca/en/about-born/). The BIS is a province-wide registry of mothers and their newborns, with 100% capture for hospital, home and birthing centre births. From April 1 2012 the BIS has collected comprehensive information on pregnancy and newborn care, including maternal demographics, health behaviors, reproductive history as well as clinical information related to pregnancy, labour and birth neonatal outcomes[11]. Pregnancies ending in miscarriage prior to 20 weeks’ gestation, and terminations of pregnancy owing to fetal anomalies at any gestational age, were excluded herein, as related information is not routinely collected in the BIS.

A diagnosis of pre-pregnancy D and H was determined using both the BIS (April 1, 2012 to March 31, 2016) and a linked (via unique health card identification number) copy of the Discharge Abstract Database (DAD) of the Canadian Institute for Health Information (CIHI) with the BIS (i.e., BIS-DAD; April 1, 2012 to March 31, 2015). The BIS-DAD contains a set of validated diagnostic codes from the International Statistical Classification of Diseases and Related Health Problems, 10th revision, Canadian version (ICD- 10-CA) for all in-hospital deliveries. Pre-pregnancy D was based on ICD-10-CA codes E10, E11, E13, E14 or O24.5-O24.7, and pre-pregnancy H was based on ICD-10-CA I10, I15, O10 or O11 (S3 Table). This BIS was supplemented with the DAD in order to apply an algorithm that allows for the highest probability of identifying all diagnoses of D and H. Gestational age in the datasets used is derived from the best clinical estimate recorded in the medical chart, largely based on ultrasound dating[12]. In Ontario, over 95% of pregnancies resulting in a birth have an ultrasound, permitting accurate pregnancy dating[13].

A diagnosis of O was defined according to the widely accepted definition of a pre-pregnancy BMI ≥ 30 kg/m^2^[14]. A pragmatic decision was made to focus on the aggregate group of obese women without further subclassification. The level of missing data for BMI in the BIS is 19%. To overcome this limitation, we identified pregnancies which could be linked to the Prenatal Screening Ontario (PSO) database to ascertain first trimester weight. The PSO database contains data for approximately 70% of pregnancies in Ontario, with some regional variation[11]. Of the 58.5% of pregnancies with a first trimester weight in the PSO database, pre-pregnancy weight was derived by subtracting the expected 2 kg weight gain to that point[15]. The missing at random (MAR) assumption was then assessed by analyzing the frequency, pattern and reason for missing pre-pregnancy BMI data. The MAR assumption cannot be verified (i.e., it is impossible to prove data are MAR rather than not missing at random (NMAR)), and therefore the plausibility of MAR could only be reasoned and hypothesized. Pre-pregnancy BMI in the BORN Information System relies on self-reported pre-pregnancy weight. Therefore, the likelihood of self-reporting pre-pregnancy weight may be driven by self-perception of whether a women’s weight adheres to societal expectations and/or comfort level to disclose perceived sensitive information. Therefore, we assumed that the probability of missing pre-pregnancy BMI depends only on pre-pregnancy weight, which would infer the data are MAR (S4 Table). Multiple imputation was then performed to generate missing pre-pregnancy BMI, using a chained equation approach based on pre-pregnancy weight in the subset of women with available pre-pregnancy weight. We created 15 imputed datasets, which were then combined across all datasets by using Rubin’s rule without data transformation, to obtain final estimates[16]. Following imputation, the level of missing for BMI was reduced by 63.6%, from 19.0% to 6.9%. Further details on how missing data were addressed can be found in S4 Table.

### Outcomes

The primary study outcome was a PTB, defined as a live birth from 24^1/7^ to 36^6/7^ weeks’ gestation. PTB was further broken down as early PTB (< 34 weeks) and late PTB (34 to 37 weeks), and piPTB (PTB following labour induction or caesarean delivery without premature rupture of membranes or other conditions indicating spontaneous onset of labour) and spPTP (all other births)[17].

In order to isolate the contribution of common factors associated with PTB, we also analyzed all PTB < 37 weeks’ gestation as follows: PTB + preeclampsia (a systolic blood pressure > 140 mm Hg or a diastolic blood pressure > 90 mmHg and new-onset proteinuria or HELLP syndrome); PTB + a large for gestational age birthweight newborn (LGA) (> 95^th^ percentile); PTB + a small for gestational age birthweight newborn (SGA) (< 5^th^ percentile), with LGA and SGA based on Canadian reference values[18].

### Analysis

Descriptive statistics summarized the proportion of pregnancies with PTB by D, O and/or H, individually and in combinations. Multivariable Poisson regression models with robust error variance generated adjusted relative risks (RR) with 95% confidence intervals (CIs), the latter adjusted for maternal age and parity. Births without D, O or H served as the reference group for all models. PAFs and 95% CIs were calculated based on the adjusted RRs, and conceptualized as the proportion of PTBs that could potentially be prevented if the exposures of D, O and/or H were eliminated[19].

To account for potentially more than one pregnancy per women within the cohort, generalized estimating equations were used in the models. Doing so did not alter the main findings. The number of pregnancies per woman is shown in S1 Table.

A two-sided p-value of <0.05 was denoted as statistically significant, and all analyses were conducted using Statistical Analysis Software Version 9.4 (Cary, NC). Ethics approval was obtained from the St. Michael’s Hospital Research Ethics Board (REB # 16–345) and the board waived the requirement for informed consent as group aggregate data was obtained from the Better Outcomes Registry and Network.

## Results

After excluding 37,827 (6.9%) births lacking information on BMI, the final cohort comprised 506,483 births. The characteristics of the cohort and the prevalence of each of the exclusive DOH states are presented in S1 Table. The non-exclusive prevalence rates for DOH have been reported earlier[2].

### PTB < 37 weeks (Fig 1, upper)

A total of 30,139 singleton pregnancies (6.0%) were complicated by PTB < 37 weeks, of which 7375 (24.5%) had D or O or H

**Fig 1:**
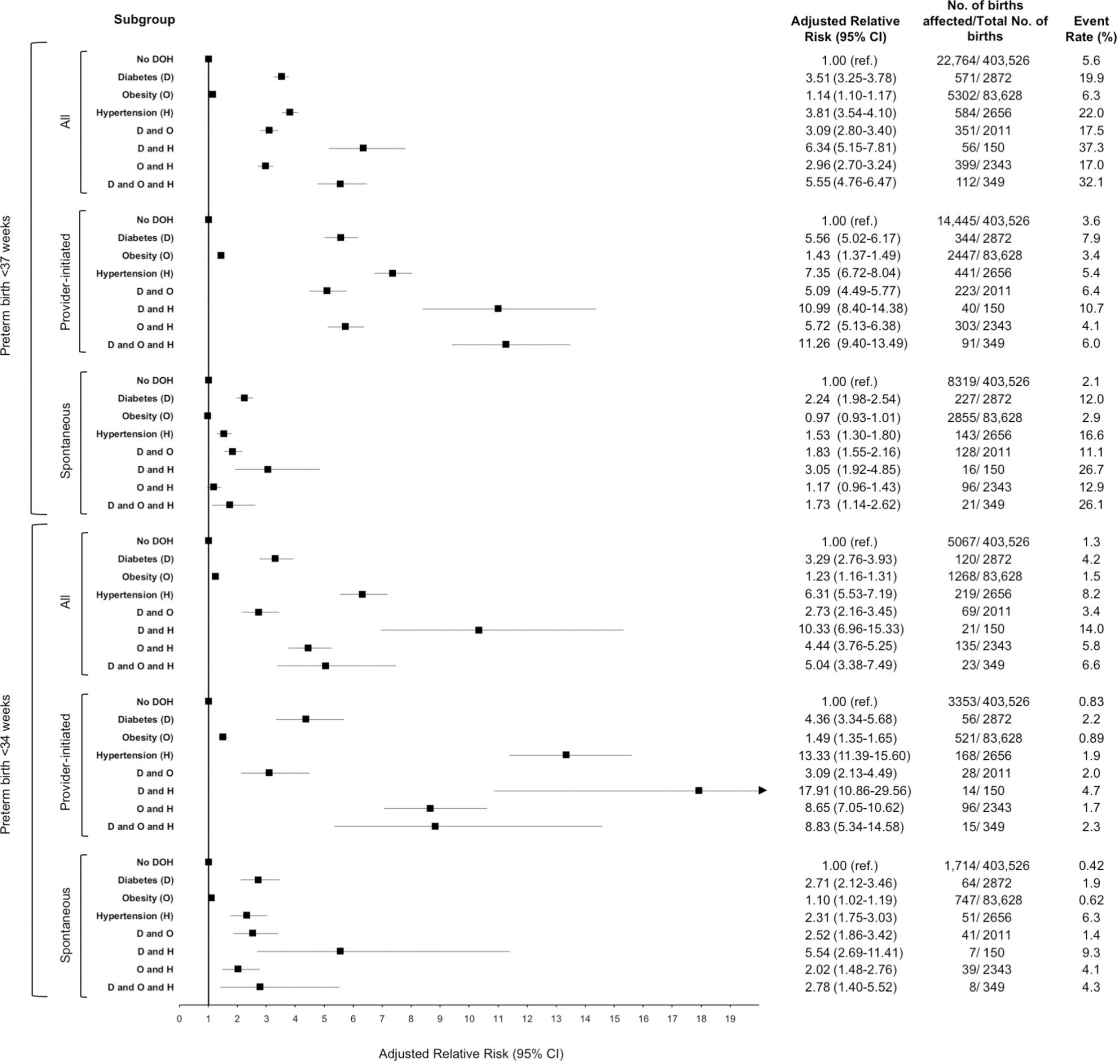
Adjusted relative risks of pre-existing diabetes, obesity and hypertension on provider initiated and spontaneous preterm birth at <37 and <34 weeks in women with a singleton birth in Ontario, between April 1, 2012 to March 31, 2016.

Women without D or O or H had a rate of PTB of 5.6%, while those with D + H had the highest rate of 37.3%. The aRR for PTB was higher for D (3.51, 95% CI 3.26–3.78) and H (3.81, 95% CI 3.55–4.10) than O (1.14, 95% CI 1.10–1.17). The combined state of DH was associated with a significantly higher aRR of PTB (6.34, 95% CI 5.14–7.80) than D or H alone. Similarly, the combined states of DO (aRR 3.09, 95% CI 2.80–3.40) and OH (aRR 2.96, 95% CI 2.70–3.24) were associated with a significant increased risk of PTB compared to the O state alone.

After stratification by piPTB versus spPTB, the risk of piPTB was significantly higher than that for spPTB. O was not found to be associated with a higher risk of spPTB (aRR 0.97, 95% CI 0.93–1.01) and only a modest increased risk of piPTB (aRR 1.35, 95% CI 1.30–1.42). However, when D or H were present with O there was a significantly higher risk of piPTB compared to the O state alone. The highest risk of piPTB was seen in women with the DH and DOH combinations (aRR 10.99, 95% CI 8.40–14.38 and aRR 11.26, 95% CI 9.40–13.49 respectively). The risk of piPTB associated with the DH state was significantly higher than for the D and H states alone.

### PTB < 34 weeks (Fig 1, lower)

A total of 6922 pregnancies (1.4%) experienced PTB < 34 weeks, of which 1855 (26.8%) were in a pregnancy affected by D, O or H. For the isolated states, the risk of PTB < 34 weeks was higher for H alone (aRR 6.31, 95% CI 5.53–7.19) than for D (aRR 3.29, 95% CI 2.76–3.93) or O alone (aRR 1.23, 95% CI 1.16–1.31). The combined state of DO (aRR 2.73, 95% CI 2.16–3.45) and OH (aRR 4.44, 95%CI 3.76–5.25) was associated with a higher risk of PTB < 34 weeks than the O state alone. The combined state of DH (aRR 10.33, 95% CI 6.96–15.33) had a higher risk than the D state alone.

The risk for PiPTB < 34 weeks was higher for the O, H or OH states than the risk of spPTB. The highest risk of piPTB < 34 weeks was seen for H (aRR 13.33, 95% CI 11.39–15.6) and DH (aRR 17.91, 95% CI 10.86–29.56).

### PTB with preeclampsia (Fig 2)

There were 962 (13.0%) pregnancies with PTB < 37 weeks’ gestation that were also affected by preeclampsia. The presence of H in isolation (aRR 45.42, 95% CI 39.69–51.99) was associated with significantly higher risk of PTB with preeclampsia than D (aRR 8.63, 95% CI 6.59–11.31) or O (aRR 1.91, 95% CI 1.68–2.16) alone. The combined state of DH (aRR 65.47, 95% CI 45.47–94.27) was associated with a higher aRR of PTB with preeclampsia than the state of D alone, while the OH state (aRR 29.60, 95% CI 25.01–35.02) was associated with a significant attenuation of the risk compared to H alone. Of the 962 cases with PTB + preeclampsia, 696 (72.4%) were piPTB. While the patterns related to DOH were similar for piPTB and spPTB, the absolute risks were higher among the former.

**Fig 2:**
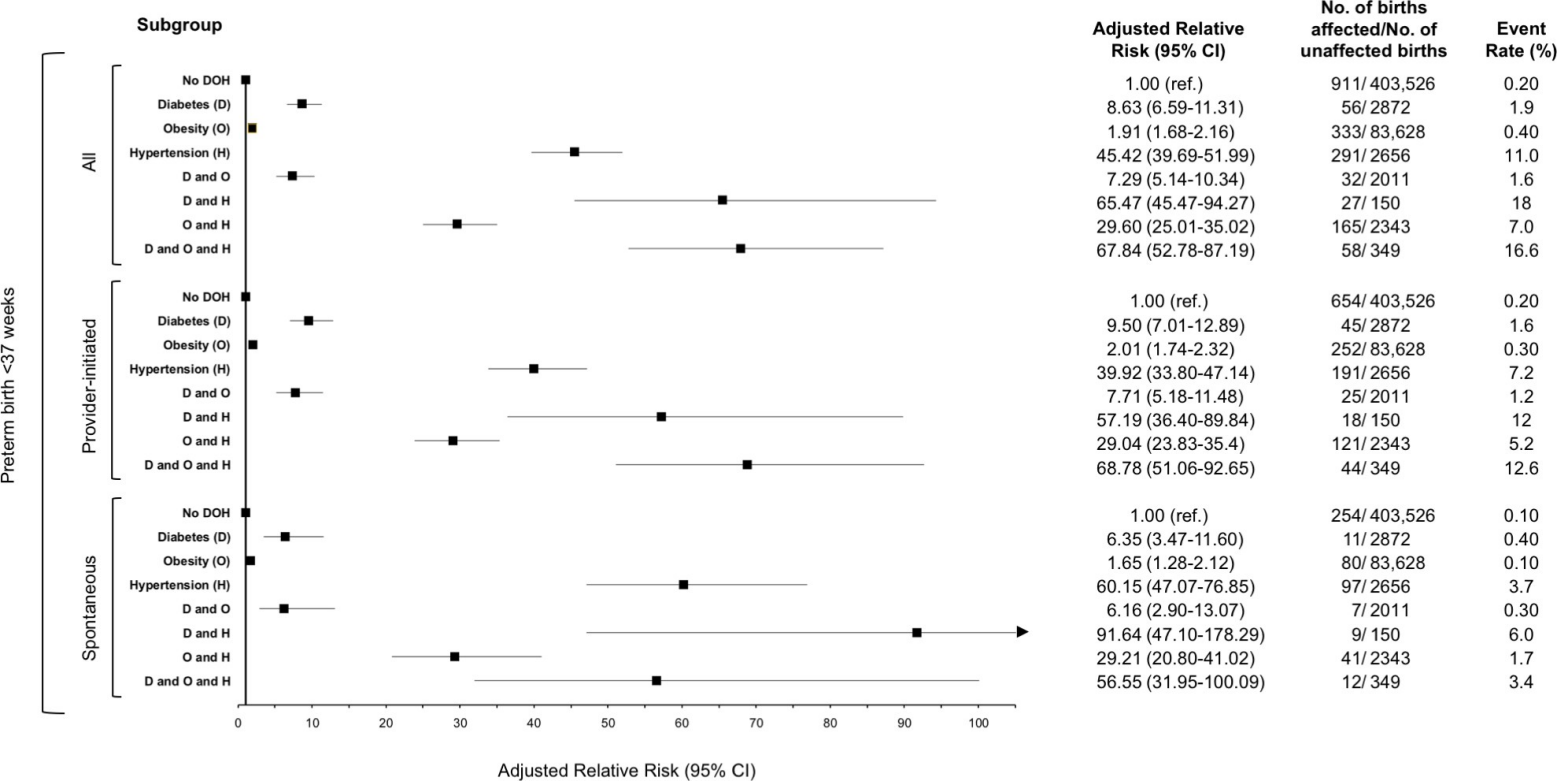
Adjusted relative risks of pre-existing diabetes, obesity and hypertension on preterm birth with preeclampsia at <37 weeks in women with a singleton birth in Ontario, between April 1, 2012 to March 31, 2016.

### PTB with SGA or LGA (Fig 3)

There were 1323 pregnancies with PTB < 37 weeks’ gestation that were also affected by newborn SGA. Of these, 410 (31.0%) were in women with D, O or H and their combinations. Only H (aRR 9.78, 95% CI 7.81–12.26) was associated with increased risk compared to the no DOH state. Of interest, when H was paired with either D or O, the aRR was less pronounced than that for H alone. Only the presence of D + O + H had a near equivalent aRR of 9.21 (95% CI 4.97–17.04).

**Fig 3:**
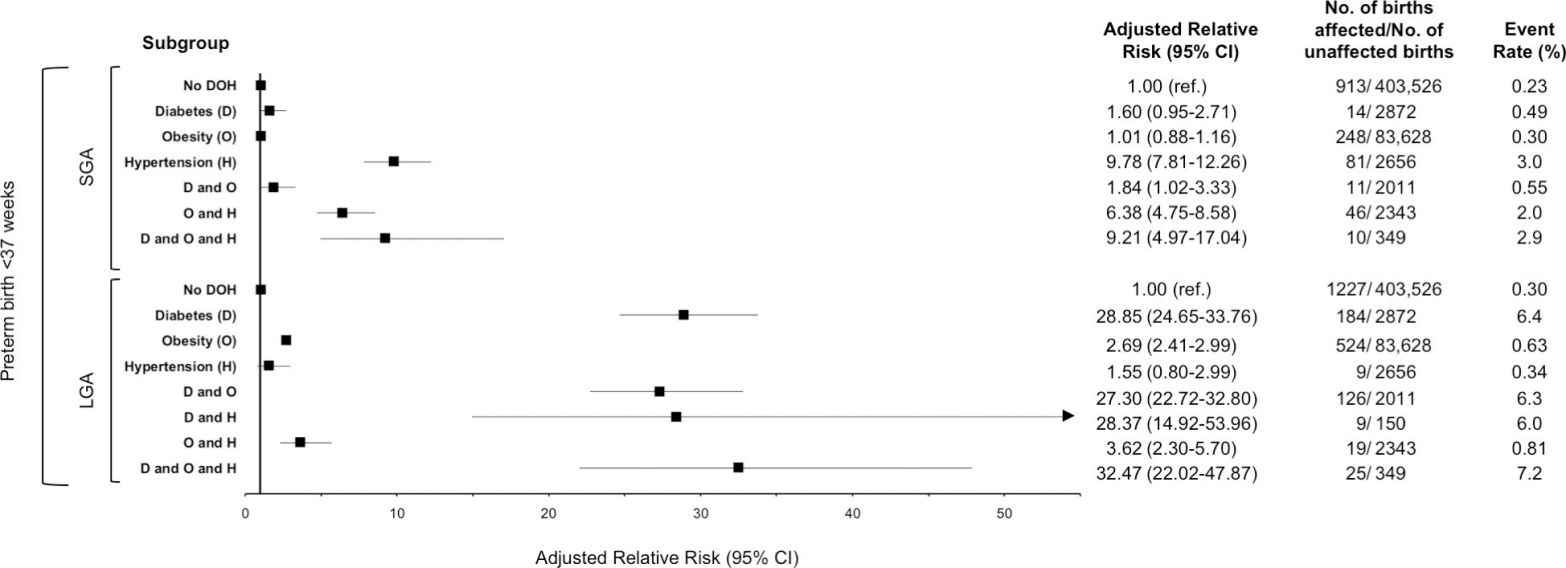
Adjusted relative risks of pre-existing diabetes, obesity and hypertension on preterm birth with small for gestational age or large for gestational age at <37 weeks in women with a singleton birth in Ontario, between April 1, 2012 to March 31, 2016. * Data for SGA in the combined D and H group was suppressed by BORN Ontario due to event rate below reporting threshold.

There were 1809 pregnancies with PTB < 37 weeks’ gestation that were also affected by a LGA neonate. Of these 896 (49.5%) were in women with D, O or H and their combinations. The highest risk of PTB + LGA were observed in women with D (aRR 28.90, 95% CI 24.65–33.76) and all combinations that include D. H was not associated with increased risk of PTB +LGA while the risk of D when combined with O was significantly higher than for O alone (Fig 3).

### PAF for PTB

The PAF for all PTB < 37 weeks was 1.36% (95% CI 1.21–1.51) for D, 2.16% (95% CI 1.29–3.02) for O, and 1.40% (95% CI 1.25–1.55) for H (S2 Table). For piPTB < 37 weeks, the highest PAF was seen for women with O (6.01%, 95% CI 5.02–7.00). For piPTB < 34 weeks, the highest PAFs were for H (5.92%, 95% CI 4.97–6.86) and O (6.54%, 95% CI 4.29–8.75).

For PTB with preeclampsia, H had an associated PAF of 15.16% (95% CI 13.5–16.79), in contrast with O (8.48%, 95% CI 6.42–10.49) or D (2.95%, 95% CI 1.88–3.42). PAFs for PTB with LGA were highest for D (9.85%, 95% CI 8.44–11.23) and O (18.22%, 95% CI 15.86–20.51), and for PTB with SGA the PAF was highest for H (4.4%, 95% CI 3.35–5.44) (S2 Table).

## Discussion

### Main findings

Pre-pregnancy D, O or H was present in nearly one quarter of women with PTB. The combined states of DH and OH, conferred a significantly higher risk of PiPTB < 37 weeks, PiPTB < 34 weeks, PTB with preeclampsia < 34 weeks and PTB with SGA than the state of D or O alone. Reductions of D, O and/or H at a population level would be expected to lead to a reduction in PTB, including PTB with concomitant abnormal fetal growth or preeclampsia.

### Strengths and limitations

This study’s strength includes its use of a large population-based sample from a validated provincial registry of all hospital births within a publicly funded health care system. This permitted generation of precise age and parity adjusted estimations of the risk of PTB in relation to combinations of D, O and H, and the calculation of PAFs representing the entire population. Additionally, access to health care is universal within this population, removing potential bias related to financially driven health care inequities. As a limitation, despite undergoing periodic validation[20], registry data can be subject to errors in coding of the exposures and outcomes, leading to misclassification bias. The latter was attenuated by using a cross-linkage and validation with the CIHI DAD. Missing data for BMI were handled by imputation[16], yet, 6.9% still had missing BMI. Furthermore, the use of this type of aggregate data does not allow for the collection of additional patient level data including the type of diabetes, adequacy and method of glycemic or hypertensive control, presence of target organ damage, blood pressure, proteinuria measurements or the criteria used to diagnose D or H. While further analysis based on subgroups stratified by these clinical factors has clinical importance, this was beyond the scope of this study that aimed mainly to provide a” birds-eye” view of the association between these common non-communicable diseases and preterm birth without unraveling the potential pathways leading to the outcome. We were also unable to adjust for potentially relevant confounders including ethnicity, socioeconomic status, geographic location or gestational weight gain due to a large degree of missing data for these variables. Despite the large number of women included in the cohort, sample size was insufficient to allow for further stratification of PTB with preeclampsia, or PTB with SGA or LGA by gestational age at delivery. Information on some historical risk factors for PTB was not available in this registry but in this mainly nulliparous cohort, their impact would be minimal and would not be likely to impact the observed increased risk of piPTB. It is recognized that in the absence of uniform indications for piPTB, we are unable to guarantee the appropriateness of the clinical decision to perform a preterm delivery. Despite this potential limitation, since decisions to end a pregnancy before 37 weeks are not taken lightly, the number of inappropriate interventions is likely small and thus not anticipated to significantly alter the results.

### Interpretation

There is a significant body of data documenting the association between D, O or H and PTB, preeclampsia, fetal growth restriction, excess fetal growth and stillbirth[4, 5, 9, 21]. The majority of these studies examined D, O or H alone, and without stratification into spontaneous versus provider initiate (formerly “iatrogenic”) preterm birth. Outside of pregnancy, there is evidence that combinations of these three risk factors can potentiate the effect exerted by each one individually[22]. The shared biological pathways of inflammation, oxidative stress, insulin resistance and mental stress [23] may explain the additive risk seen when D, O and/or H arise together.

O affects over a third of women of reproductive age in the US[24]. Pre-pregnancy O is seen in nearly 25% of gravid women in the US[25] and 17% of those in Canada[26,27]. A meta-analysis of 1 095 834 women found no overall increased risk of PTB in overweight and obese women (RR 1.06; 95% CI 0.87 to 1.30), but a heightened risk of piPTB (RR 1.30; 95%CI 1.23 to 1.37)[28]. A more recent metanalysis of individual patient data examining the relationship of maternal BMI and gestational weight gain to pregnancy complications revealed, similar to our study, an increased risk of PTB in women with a BMI > 30 (aRR 1.33, 95% CI 1.25–1.41). This study was able to adjust for maternal age, education level, parity and smoking status but unlike our study, did not stratify by early versus late PTB or piPTB versus spPTB[29]. In a Swedish study of the relation between BMI and PTB among 1 599 551 deliveries, elevated BMI was associated with a higher risk of PTB only among women with pre-pregnancy D and H[8]. This supports our current findings that, in obese women, D, and to a greater extent H, seem to be the primary drivers of piPTB.

The incidence of both diabetes and chronic hypertension is on the rise and this includes a rise in the incidence of hypertension complicated by diabetes [30]. The impact of these individual conditions on perinatal adverse outcomes is also well known. Similar to our findings, a recent prospective cohort study demonstrated an independent increased risk of preeclampsia, piPTB and SGA <5% in women with hypertension after adjusting for maternal weight, preexisting diabetes and other baseline characteristics. In contrast with our results, they did not find an increased risk of spPTB [22], possibly due to the known deficiencies that exist with regards to the accuracy of PTB classification in large administrative databases [31]. Generalizability of our results can be further inferred by the results of a Finnish population based study that demonstrated a strong interaction between maternal BMI and maternal diabetes on offspring prematurity but similar to the results of our study, revealed no additive effect of increased maternal BMI to the inherent high risk of prematurity in women with type 2 diabetes or diabetes treated with insulin [32].

Sub classifying PTB into piPTB and spPTB is important, from both a clinical and epidemiological standpoint, as etiology and prevention strategies for each differ. For spPTB, which is of a multifactorial and poorly understood etiology, the current mainstay of prevention in women with historical risk factors or a risk factor that develops during the pregnancy (e,g cervical shortening) is maternal progesterone therapy or mechanical intervention, such as cervical cerclage. For piPTB, which is largely mediated by preeclampsia, SGA, or both, prophylactic low dose aspirin reduces preterm preeclampsia by 40% in women at higher risk[33]. Identifying women who can benefit the most from ASA is important. Complex risk stratification strategies have been employed in clinical studies but are difficult to implement in routine clinical practice. Even after the publication of national guidelines on prophylactic ASA use were published, actual uptake remained low [34]. The findings from the current study might encourage health care providers, as well as policy makers, to focus piPTB prevention strategies, including low dose ASA, on women at highest risk, especially those with DOH. Although the efficacy of lifestyle interventions in obese pregnant women is limited [35–37], our finding that the highest population attributable fraction for PTB < 34 weeks (3.42%) and PTB +LGA (18.22%) was seen in obese women has potential clinical relevance as it identifies a population subset that might preferentially benefit from a focused intervention aimed at PTB reduction. The PAFs and relative risks we describe in this study highlight the dilemma that we face as health care providers in the quest to reduce the rate of PTB. Do we focus complex interventions and surveillance strategies on those at highest individual risk or rather apply broader interventions that could have a larger population-based impact? Our results suggest that a combination of both strategies is likely warranted.

## Conclusion

In conclusion, combinations of D, O and H significantly magnify the risk of PTB, especially piPTB, and PTB with altered fetal growth or preeclampsia. These data may inform health policy and clinical decision-making around the prevention of PTB and related adverse outcomes, both at the population- and patient level. Further studies are needed to identify the most effective PTB reduction interventions in women with DOH.

## Supporting information

S1 Table Characteristics of singleton pregnancies in Ontario, April 1, 2012 to March 31, 2016. All data are presented as a number (%) unless otherwise indicated. Data are suppressed in instances where a cell count is less than 6.
(DOCX)

S2 Table Population attributable fractions (PAF) of pre-existing diabetes, obesity and hypertension on preterm delivery in women who had a singleton birth in Ontario, April 1, 2012 to March 31, 2016. All data are presented as a %. Data are suppressed in instances where a cell count is less than 6.
(DOCX)

S3 Table International Classification of Diseases 10th (ICD-10) revision codes. (DOCX)

S4 Table Addressing missing data. (DOCX)
