# Supplementary material for: Impact of diabetes, obesity and hypertension on preterm birth: Population-based study
Source: PLoS One. 2020 Mar 25;15(3):e0228743. doi: 10.1371/journal.pone.0228743 (PMC7094836; doi:10.1371/journal.pone.0228743)
Supplement: S1 Table — All data are presented as a number (%) unless otherwise indicated. Data are suppressed in instances where a cell count is less than 6. (DOCX) [file pone.0228743.s001.docx]

| **Table S1:** Characteristics of singleton pregnancies in Ontario, April 1, 2012 to March 31, 2016 based on exclusive D, O and H states individually, and in all exclusive combinations. All data are presented as a number (%) unless otherwise indicated. Data are suppressed in instances where a cell count is less than 6.  **Measurement** | | | | | | | | | | | | | | |
| --- | --- | --- | --- | --- | --- | --- | --- | --- | --- | --- | --- | --- | --- | --- |
| **Characteristic** | **Diabetes (D)**  (N = 2,872) | **Obesity (O)**  (N = 83,628) | **Hypertension (H)**  (N = 2,656) | | **D and O**  (N = 2,011) | | **D and H**  (N = 150) | | **O and H**  (N = 2,343) | | **D and O and H**  (N = 349) | |  |  |
|  | 0.57% | 16.51% | 0.52% | | 0.40% | | 0.03% | | 0.46% | | 0.07% | |  |  |
| **Maternal age** (years) |  |  |  | |  | |  | |  | |  | |  |  |
| Mean ± SD | 32.00 ± 5.63 | 30.53 ± 5.32 | | 33.31 ± 5.53 | | 32.53 ± 5.26 | | 35.19 ± 6.00 | | 33.41 ± 5.29 | | 34.23 ± 4.94 | |  |
| ≤ 19 | 43 (1.5) | 1,480 (1.8) | | 26 (1.0) | | 10 (0.50) | | <6 (S) | | 9 (0.40) | | <6 (S) | |  |
| 20-24 | 242 (8.4) | 9,913 (11.9) | | 130 (4.9) | | 131 (6.5) | | <6 (S) | | 103 (4.4) | | 9 (2.6) | |  |
| 25-29 | 631 (22.0) | 23,650 (28.3) | | 486 (18.3) | | 436 (21.7) | | 14 (9.3) | | 444 (19.0) | | 46 (13.2) | |  |
| 30-34 | 996 (34.7) | 29,086 (34.8) | | 874 (32.9) | | 707 (35.2) | | 45 (30.0) | | 785 (33.5) | | 111 (31.8) | |  |
| 35-39 | 698 (24.3) | 15,753 (18.8) | | 791 (29.8) | | 539 (26.8) | | 45 (30.0) | | 696 (29.7) | | 132 (37.8) | |  |
| ≥ 40 | 262 (9.1) | 3,746 (4.5) | | 349 (13.1) | | 188 (9.3) | | 39 (26.0) | | 306 (13.1) | | 49 (14.0) | |  |
| **Maternal Ethnicity** |  |  |  | |  | |  | |  | |  | |  |  |
| Caucasian | 1,072 (37.3) | 36,593 (43.8) | | 1,041 (39.2) | | 776 (38.6) | | 52 (34.7) | | 1,018 (43.4) | | 139 (39.8) | |  |
| Asian | 770 (26.8) | 6,170 (7.4) | | 619 (23.3) | | 267 (13.3) | | 49 (32.7) | | 227 (9.7) | | 40 (11.5) | |  |
| Black | 126 (4.4) | 5,292 (6.3) | | 232 (8.7) | | 155 (7.7) | | 10 (6.7) | | 295 (12.6) | | 33 (9.5) | |  |
| Other | 147 (5.1) | 3,177 (3.8) | | 94 (3.5) | | 140 (7.0) | | 8 (5.3) | | 81 (3.5) | | 31 (8.9) | |  |
| Missing | 757 (26.4) | 32,396 (38.7) | | 670 (25.2) | | 673 (33.5) | | 31 (20.7) | | 722 (30.8) | | 106 (30.4) | |  |
| **Parity** |  |  |  | |  | |  | |  | |  | |  |  |
| Nulliparous | 1,155 (40.2) | 31,006 (37.1) | | 1,177 (44.3) | | 682 (33.9) | | 71 (47.3) | | 945 (40.3) | | 138 (39.5) | |  |
| Parous | 1,684 (58.6) | 51,982 (62.2) | | 1,435 (54.0) | | 1,320 (65.6) | | 78 (52.0) | | 1,378 (58.8) | | 203 (58.2) | |  |
| Unknown | 33 (1.1) | 640 (0.80) | | 44 (1.7) | | 9 (0.40) | | <6 (S) | | 20 (0.90) | | 8 (2.3) | |  |
| **Maternal body mass index, kg/m2** (mean ± SD) | 24.42 ± 3.25 | 35.48 ± 5.63 | 24.67 ± 3.24 | | 36.8 ± 6.02 | | 25.98 ± 2.71 | | 37.2 ± 6.13 | | 38.33 ± 5.95 | |  |  |
| **Gestational age at delivery** (weeks) |  |  |  | |  | |  | |  | |  | |  |  |
| ≤ 22 | 7 (0.20) | 212 (0.30) | 6 (0.20) | | <6 (S) | | 0 (0) | | 11 (0.50) | | <6 (S) | |  |  |
| 23-31 | 56 (1.9) | 883 (1.1) | 156 (5.9) | | 33 (1.6) | | 11 (7.3) | | 93 (4.0) | | 15 (4.3) | |  |  |
| 32-36 | 539 (18.8) | 4,671 (5.6) | 447 (16.8) | | 337 (16.8) | | 46 (30.7) | | 322 (13.7) | | 101 (28.9) | |  |  |
| 37-42 | 2,265 (78.9) | 77,797 (93.0) | 2,045 (77.0) | | 1,633 (81.2) | | 93 (62.0) | | 1,915 (81.7) | | 232 (66.5) | |  |  |
| ≥ 43 | <6 (S) | 56 (0.10) | <6 (S) | | <6 (S) | | 0 (0) | | <6 (S) | | 0 (0) | |  |  |
| Missing | 0 (0) | 9 (0) | 0 (0) | | 0 (0) | | 0 (0) | | 0 (0) | | 0 (0) | |  |  |
| **Number of pregnancies per woman**^1^ |  |  |  | |  | |  | |  | |  | |  |  |
| 1 | 4,877 (88.8) | 76,909 (85.3) | 5,144 (90.8) | | 2,130 (88.4) | | 462 (90.1) | | 2,491 (90.0) | | 323 (90.0) | |  |  |
| 2 | 600 (10.9) | 12,811 (14.2) | 511 (9.0) | | 272 (11.3) | | 49 (9.6) | | 271 (9.8) | | 35 (9.7) | |  |  |
| ≥3 | 16 (0.30) | 457 (0.50) | 12 (0.20) | | 7 (0.30) | | <6 (S) | | 6 (0.20) | | <6 (S) | |  |  |

^1^During study period from April 1, 2012 to March 31, 2016
