# Supplementary material for: Impact of diabetes, obesity and hypertension on preterm birth: Population-based study
Source: PLoS One. 2020 Mar 25;15(3):e0228743. doi: 10.1371/journal.pone.0228743 (PMC7094836; doi:10.1371/journal.pone.0228743)
Supplement: S2 Table — All data are presented as a %. Data are suppressed in instances where a cell count is less than 6. (DOCX) [file pone.0228743.s002.docx]

| **Table S2:** Population attributable fractions (PAF) of pre-existing diabetes, obesity and hypertension on preterm delivery in women who had a singleton birth in Ontario, April 1, 2012 to March 31, 2016. All data are presented as a %. Data are suppressed in instances where a cell count is less than 6.  **Measurement** | | | | | | | | | | | | |
| --- | --- | --- | --- | --- | --- | --- | --- | --- | --- | --- | --- | --- |
| **Characteristic** | **Diabetes (D)**  (N = 2,872) | **Obesity (O)**  (N = 83,628) | | **Hypertension (H)**  (N = 2,656) | **D and O**  (N = 2,011) | **D and H**  (N = 150) | | **O and H**  (N = 2,343) | | **D and O and H**  (N = 349) |  |  |
| **Preterm delivery 24 to <37 weeks** | 1.36 (1.21-1.51) | | 2.16 (1.29-3.02) | 1.40 (1.25-1.55) | 0.81 (0.69-0.93) | | 0.17 (0.12-0.22) | | 0.86 (0.74-0.98) | 0.33 (0.26-0.40) | |  |
| Spontaneous | 0.72 (0.56-0.88) | | -0.49 (-1.89-0.89) | 0.28 (0.12-0.43) | 0.32 (0.19-0.44) | | 0.07 (0.02-0.11) | | 0.07 (-0.09-0.24) | 0.04 (-0.01-0.09) | |  |
| Provider-initiated | 2.30 (2.01-2.59) | | 6.01 (5.02-7.00) | 3.11 (2.78-3.44) | 1.45 (1.21-1.68) | | 0.27 (0.18-0.37) | | 2.06 (1.79-2.34) | 0.64 (0.49-0.79) | |  |
| **Preterm delivery 24 to <34 weeks** | 1.18 (0.89-1.48) | | 3.42 (1.82-5.00) | 2.69 (2.28-3.10) | 0.63 (0.40-0.86) | | 0.27 (0.14-0.40) | | 1.55 (1.22-1.87) | 0.24 (0.11-0.37) | |  |
| Spontaneous | 0.95 (0.59-1.30) | | 1.57 (-0.75-3.85) | 0.68 (0.36-1.00) | 0.6 (0.31-0.90) | | 0.16 (0.03-0.30) | | 0.45 (0.16-0.74) | 0.13 (0.00-0.26) | |  |
| Provider-initiated | 1.62 (1.08-2.16) | | 6.54 (4.29-8.75) | 5.92 (4.97-6.86) | 0.74 (0.35-1.14) | | 0.47 (0.20-0.74) | | 3.27 (2.55-3.99) | 0.53 (0.24-0.83) | |  |
| **Preeclampsia + Preterm delivery 24 to <37 weeks** | 2.65 (1.88-3.42) | | 8.48 (6.42-10.49) | 15.16 (13.50-16.79) | 1.47 (0.88-2.05) | | 1.38 (0.85-1.91) | | 8.50 (7.21-9.78) | 3.05 (2.27-3.84) | |  |
| Provider-initiated | 2.95 (2.00-3.90) | | 9.4 (6.98-11.75) | 13.75 (11.86-15.59) | 1.65 (0.93-2.38) | | 1.28 (0.67-1.88) | | 8.69 (7.15-10.21) | 3.25 (2.29-4.20) | |  |
| Spontaneous | 1.85 (0.58-3.11) | | 6.18 (1.91-10.28) | 18.68 (15.20-22.03) | 1.17 (0.16-2.18) | | 1.78 (0.62-2.93) | | 7.73 (5.34-10.05) | 2.26 (0.95-3.55) | |  |
| **LGA95 + Preterm delivery 24 to <37 weeks** | 9.85 (8.44-11.23) | | 18.22 (15.86-20.51) | 0.18 (-0.26-0.61) | 6.74 (5.56-7.91) | | 0.48 (0.16-0.81) | | 0.80 (0.32-1.27) | 1.36 (0.81-1.90) | |  |
| **SGA5 + Preterm delivery 24 to <37 weeks** | 0.34 (-0.24-0.91) | | 0.15 (-4.24-4.35) | 4.40 (3.35-5.44) | 0.32 (-0.14-0.78) | | - | | 2.36 (1.56-3.15) | 0.53 (0.16-0.91) | |  |
