# Supplementary material for: Impact of diabetes, obesity and hypertension on preterm birth: Population-based study
Source: PLoS One. 2020 Mar 25;15(3):e0228743. doi: 10.1371/journal.pone.0228743 (PMC7094836; doi:10.1371/journal.pone.0228743)
Supplement: S3 Table — (DOCX) [file pone.0228743.s003.docx]

**Table S3.** International Classification of Diseases 10^th^ (ICD-10) Revision Codes

|  | **Includes** | **Excludes** |
| --- | --- | --- |
| **E10** Insulin-dependent diabetes mellitus | Diabetes (mellitus):   - brittle - juvenile-onset - ketosis-prone - type I | Diabetes mellitus (in):   - malnutrition-related (E12.-) - neonatal (P70.2) - pregnancy, childbirth and the puerperium (O24.-)   Glycosuria:   - NOS (R81) - renal (E74.8)   Impaired glucose tolerance (R73.0)  Postsurgical hypoinsulinaemia (E89.1) |
| **E11** Non-insulin-dependent diabetes mellitus  *[See before E10 for subdivisions]* | Diabetes (mellitus)(nonobese)(obese):   - adult-onset - maturity-onset - nonketotic - stable - type II   Non-insulin-dependent diabetes of the young | Diabetes mellitus (in):   - malnutrition-related (E12.-) - neonatal (P70.2) - pregnancy, childbirth and the puerperium (O24.-)   Glycosuria:   - NOS (R81) - renal (E74.8)   Impaired glucose tolerance (R73.0)  Postsurgical hypoinsulinaemia (E89.1) |
| **E13** Other specified diabetes mellitus  *[See before E10 for subdivisions]* |  | Diabetes mellitus (in):   - insulin-dependent (E10.-) - malnutrition-related (E12.-) - neonatal (P70.2) - non-insulin-dependent (E11.-) - pregnancy, childbirth and the puerperium (O24.-)   Glycosuria:   - NOS (R81) - renal (E74.8)   Impaired glucose tolerance (R73.0)  Postsurgical hypoinsulinaemia (E89.1) |
| **E14** Unspecified diabetes mellitus  [See before E10 for subdivisions] | Diabetes NOS | Diabetes mellitus (in):   - insulin-dependent (E10.-) - malnutrition-related (E12.-) - neonatal (P70.2) - non-insulin-dependent (E11.-) - pregnancy, childbirth and the puerperium (O24.-)   Glycosuria:   - NOS (R81) - renal (E74.8)   Impaired glucose tolerance (R73.0)  Postsurgical hypoinsulinaemia (E89.1) |
| **I10** Essential (primary) hypertension | High blood pressure  Hypertension (arterial)(benign)(essential)  (malignant)(primary)(systemic) | Involving vessels of:   - brain (I60-I69) - eye (H35.0) |
| **I15** Secondary hypertension |  | Involving vessels of:   - brain (I60-I69) - eye (H35.0) |
| **O10** Pre-existing hypertension complicating pregnancy, childbirth and the puerperium^1-6^ | The listed conditions with pre-existing proteinuria | That with increased or superimposed proteinuria |
| **O11** Pre-existing hypertensive disorder with superimposed proteinuria | Conditions in O10.- complicated by increased proteinuria  Superimposed pre-eclampsia |  |
| **O24** Diabetes mellitus in pregnancy^7-10^ | Diabetes in childbirth and the puerperium  An additional code from the range of E10-E14 may also be selected to further specify any existing complications of diabetes. |  |

Source: https://icd.who.int/

Notes:

1. **O10.0 Pre-existing essential hypertension complicating pregnancy, childbirth and the puerperium:** Any condition in I10 specified as a reason for obstetric care during pregnancy, childbirth or the puerperium
2. **O10.1 Pre-existing hypertensive heart disease complicating pregnancy, childbirth and the puerperium:** Any condition in I11.- specified as a reason for obstetric care during pregnancy, childbirth or the puerperium
3. **O10.2 Pre-existing hypertensive renal disease complicating pregnancy, childbirth and the puerperium:** Any condition in I12.- specified as a reason for obstetric care during pregnancy, childbirth or the puerperium
4. **O10.3 Pre-existing hypertensive heart and renal disease complicating pregnancy, childbirth and the puerperium:** Any condition in I13.- specified as a reason for obstetric care during pregnancy, childbirth or the puerperium
5. **O10.4 Pre-existing secondary hypertension complicating pregnancy, childbirth and the puerperium:** Any condition in I15.- specified as a reason for obstetric care during pregnancy, childbirth or the puerperium
6. **O10.9 Unspecified pre-existing hypertension complicating pregnancy, childbirth and the puerperium**
7. **024.5 Pre-existing type I diabetes mellitus in pregnancy**
8. **024.6 Pre-existing type 2 diabetes mellitus in pregnancy**
9. **024.7 Pre-existing diabetes mellitus of other or unspecified type in pregnancy**
10. **024.8 Diabetes mellitus arising in pregnancy (gestational)** Includes diabetes mellitus in pregnancy, unspecified
