# Supplementary material for: Impact of diabetes, obesity and hypertension on preterm birth: Population-based study
Source: PLoS One. 2020 Mar 25;15(3):e0228743. doi: 10.1371/journal.pone.0228743 (PMC7094836; doi:10.1371/journal.pone.0228743)
Supplement: S4 Table — (DOCX) [file pone.0228743.s004.docx]

**Table S4.** Addressing missing data

| **Recommendation** | **How was this addressed?** |
| --- | --- |
| We advise that authors report the number of missing values for each variable of interest (exposures, outcomes, confounders) and for each step in the analysis | The level of missing of pre-pregnancy BMI prior to imputation and following imputation is summarized in the following, “Following imputation, the level of missing for pre-pregnancy BMI was reduced by 63.6%, from 19.0% to 6.9%.” |
| Authors should give reasons for missing values if possible and indicate how many individuals were excluded because of missing data when describing the flow of participants through the study (see also item 13). | The only patients excluded from this birth cohort were those with missing BMI following imputation (37,827; 6.9%) and this is reported in the results section.  Reasons for missing pre-pregnancy BMI are summarized in the following, “Pre-pregnancy BMI in the BORN Information System relies on self-reported pre-pregnancy weight. Therefore, the likelihood of self-reporting pre-pregnancy weight may be driven by self-perception of whether a women’s weight adheres to societal expectations and/or comfort level to disclose sensitive information. Therefore, we assumed that the probability of missing pre-pregnancy BMI depends only on pre-pregnancy weight, which would infer the data are MAR.” |
| For analyses that account for missing data, authors should describe the nature of the analysis (e.g., multiple imputation) and the assumptions that were made (e.g., missing at random, see Box 6)”. | The rationale behind why the MAR is plausible is summarized in the following, “The MAR assumption cannot be verified (i.e., it is impossible to prove data are MAR rather than not missing at random (NMAR)), and therefore the plausibility of MAR can only be reasoned and hypothesized(1). Pre-pregnancy BMI in the BORN Information System relies on self-reported pre-pregnancy weight. Therefore, the likelihood of self-reporting pre-pregnancy weight may be driven by self-perception of whether a women’s weight adheres to societal expectations and/or comfort level to disclose perceived sensitive information. Therefore, we assumed that the probability of missing pre-pregnancy BMI depends only on pre-pregnancy weight, which would infer the data are MAR.”  The multiple imputation procedure is summarized in the following, “Multiple imputation was then performed to generate missing pre-pregnancy BMI using a chained equation approach based on pre-pregnancy weight in the subset of women with available pre-pregnancy weight. We created 15 imputed datasets, which were then combined across all datasets by using Rubin’s rule to obtain final ~~e~~stimates(14).” |
| Box 6 - Results using any of these approaches should be compared with those from complete case analyses, and important differences discussed. The plausibility of assumptions made in missing data analyses is generally unverifiable. In particular it is impossible to prove that data are MAR, rather than MNAR. Such analyses are therefore best viewed in the spirit of sensitivity analysis (see items 12e and 17). | A comparison of estimates from a complete case analysis vs. multiple imputation was not performed.  We implemented a method whereby in a subset of women with non-missing pre-pregnancy BMI, we randomly assigned missing pre-pregnancy BMI to an equivalent number of participants with true missing pre-pregnancy BMI (i.e., the number of patients where pre-pregnancy BMI would be imputed). We then applied our imputation procedure to impute missing pre-pregnancy BMI and assessed the level of agreement between the imputed pre-pregnancy BMI and actual pre-pregnancy BMI via the R^2^ statistic. An R^2^ value of approximately 0.75 was achieved, indicating good agreement. |

1. Vandenbroucke, J. P., Von Elm, E., Altman, D. G., Gøtzsche, P. C., Mulrow, C. D., Pocock, S. J., ... & Strobe Initiative. (2007). Strengthening the Reporting of Observational Studies in Epidemiology (STROBE): explanation and elaboration. PLoS medicine, 4(10), e297.
